# Application of TRPS1 in ER-negative or low expression distant metastatic breast carcinoma

**DOI:** 10.3389/pore.2025.1612138

**Published:** 2025-08-01

**Authors:** Runze Zhang, Jing Liu, Lei Jiang, Zhiqiang Lang

**Affiliations:** Yantai Yuhuangding Hospital, Yantai, China

**Keywords:** breast cancer, distant metastatic breast carcinomas, ER, TRPS1, GATA3

## Abstract

**Purpose:**

Traditional markers have various limitations in recognizing the breast origin of distant metastatic breast carcinoma (DMBC), especially in ER-negative or low expression cases. In recent years, TRPS1 has been reported as a breast marker with satisfactory sensitivity and specificity in triple-negative breast cancers (TNBC). We aimed to compare the expression of TRPS1, GATA3, and GCDFP-15 in ER-negative or low-ER-expressing DMBC, and to further evaluate the diagnostic value of TRPS1.

**Methods:**

Immunohistochemical staining for TRPS1, GATA3, and GCDFP-15 was performed in 107 cases of ER-negative or low expression DMBC specimens. Nuclear staining was considered positive for TRPS1 and GATA3, and cytoplasmic staining was considered positive for GCDFP-15.

**Results:**

The positive rates for TRPS1, GATA3, and GCDFP-15 were 90.65% (97/107), 91.59% (98/107), and 42.99% (46/107), respectively. There was no significant difference in the expression rate and intensity between the first two markers (*p* = 0.929), but both rates were significantly higher than that of GCDFP-15 (*p* < 0.05). Among these, 6 cases showed positive expression for TRPS1 while GATA3 and GCDFP-15 were negative; 8 cases showed positive expression for GATA3 while TRPS1 and GCDFP-15 were negative.

**Conclusion:**

TRPS1 is as effective as GATA3 in confirming breast origin for ER-negative or low expression DMBC, and the two markers exhibit excellent complementary effects, both outperforming GCDFP-15. The combined application of TRPS1 and GATA3 is the optimal method to confirm that ER-negative or low-expression distant metastatic carcinoma originates from the breast.

## Introduction

Breast cancer is the most common malignant tumor in women, exhibiting a high rate of axillary lymph node metastasis with distant metastasis also commonly observed. When metastasis occurs, identifying the origin and immunophenotype of the metastatic lesions is crucial for treatment. Currently, there are many relatively specific immunohistochemical markers or combinations used for the auxiliary diagnosis of breast cancer, including GATA3, mammaglobin, GCDFP-15, and ER, similar to how TTF-1 and NapsinA are used for diagnosing lung cancer, CDX2 for gastrointestinal-origin cancers, TG for thyroid cancer, Hepatocyte and Arg-1 for liver cancer, and PSA and AR for prostate cancer, etc. However, these so-called site-specific markers are not highly specific, and abnormal expression can occur in rare cases. For example, TTF-1 and CDX2 can also show positive rates of 4.6% and 1.8% in breast cancer respectively [1]. The same situation also occurs in breast cancer. For example, GATA3 can also be highly expressed in urothelial carcinoma, parathyroid tumors, adnexal tumors, and certain germ cell tumors, etc. [2]. ER can also be expressed in thyroid papillary carcinoma, lung cancer, and digestive system cancers [3–5]. Mammaglobin and GCDFP-15, however, have the issue of low sensitivity. Therefore, although most distant metastatic breast carcinomas (DMBC) can be confirmed as breast-origin by these traditional immunohistochemical markers, they sometimes provide insufficient evidence, posing a challenge for definitive diagnosis. This is especially true for ER-negative or low-expression cases, as well as tumors where the ER expression changes after distant metastasis of the primary lesion. In recent years, a new marker, trichorhinophalangeal syndrome type 1, also known as transcriptional repressor GATA binding 1 (TRPS1), has been found to be a breast cancer marker with high specificity and sensitivity. It is associated with the occurrence and development of various malignant tumors and its molecular mechanism and prognostic impact on breast cancer have also been confirmed [6–9]. Studies have shown that the positive rate of TRPS1 in ER-positive breast cancer is 98%, similar to GATA3 (96%), while the positive rate in triple-negative breast cancer (TNBC) is 86%, significantly higher than GATA3 (45%) [10]. Other studies also support the high sensitivity and specificity of TRPS1 for TNBC, including a higher proportion of expression in male breast cancer, metaplastic breast cancer, and lymph node metastasis [11–15]. TRPS1 also showed high specificity and sensitivity in metastatic cytological samples [16, 17]. Its value in the differential diagnosis of mammary and extramammary Paget’s disease and melanoma *in situ* has also been confirmed [18, 19]. However, its application value in distant metastatic cases, particularly in ER-negative or low-expression DMBC, has not been fully studied. This study explores the practical utility of TRPS1 by comparing the expression of TRPS1, GATA3, and GCDFP-15 in ER-negative or low-expression DMBC.

## Materials and methods

A total of 107 cases of ER-negative or low-expression DMBC were collected from Yantai Yuhuangding Hospital between January 2021 and November 2023. Immunohistochemical staining for TRPS1, GATA3, and GCDFP-15 was performed, including 91 cases with ER-negative expression and 16 cases with ER-low expression (positive rate 1%–10%). The samples included core needle biopsies, whole-tissue sections, and cytological specimens. The metastatic sites included cervical lymph nodes (41 cases), lung (20 cases), liver (12 cases), axilla (8 cases without a concurrent primary tumor), skin (5 cases), supraclavicular lymph nodes (5 cases), pleural effusion cell blocks (5 cases), bronchial biopsy tissue (2 cases), pleura (1 case), brain (1 case), infraclavicular lymph nodes (1 case), sacrum (1 case), right upper abdomen (1 case), ilium (1 case), kidney (1 case), fallopian tube (1 case), and ascitic fluid cell blocks (1 case). TRPS1 rabbit monoclonal antibody (1:3000, EPR16171, Abcam, Cambridge, UK), GATA3 Rabbit monoclonal antibody (1:500, EPR16651, Abcam, Cambridge, UK) and GCDFP-15 Rabbit monoclonal antibody (1:100, EPR1582, Abcam, Cambridge, UK) were used for immunohistochemical staining. TRPS1 and GATA3 nuclear staining were considered positive, and GCDFP-15 cytoplasmic staining was considered positive. The semi-quantitative scoring method was used, with categories of negative (<1%), weakly positive (1%–10%), moderately positive (11%–50%), and strongly positive (>50%). Immunohistochemical (IHC) staining was performed using the Roche autostainer system (BenchMark ULTRA PLUS) following standard automated protocols, as previously described [10]. Statistical analysis: The associations between TRPS1, GATA3, and GCDFP-15 expression in ER-negative or low-expression DMBC were analyzed by Wilcoxon Signed-Rank Test and McNemar’s Test. The level of significance was set at 0.05.

## Results

**FIGURE 1:**
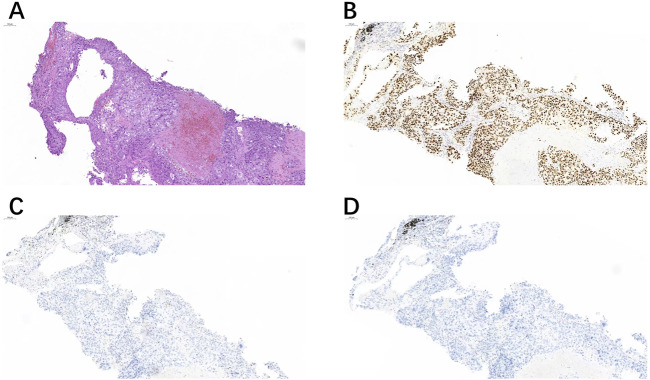
A right cervical lymph node biopsy **(A)** showed positive expression of TRPS1 **(B)**, but negative expression of GATA3 **(C)** and GCDFP-15 **(D)**.

**FIGURE 2:**
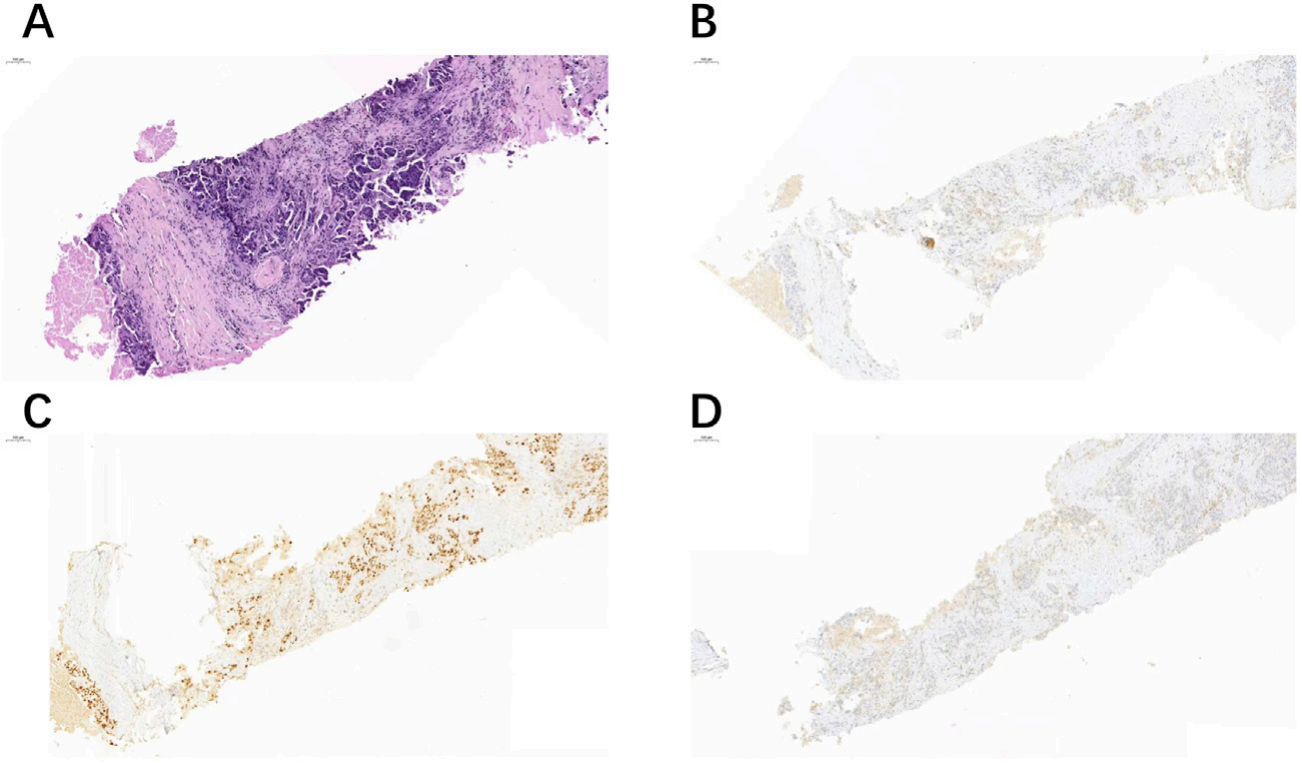
Another case of right cervical lymph node biopsy **(A)** showed positive expression of GATA3 **(C)**, but negative expression of TRPS1 **(B)** and GCDFP-15 **(D)**.

**FIGURE 3:**
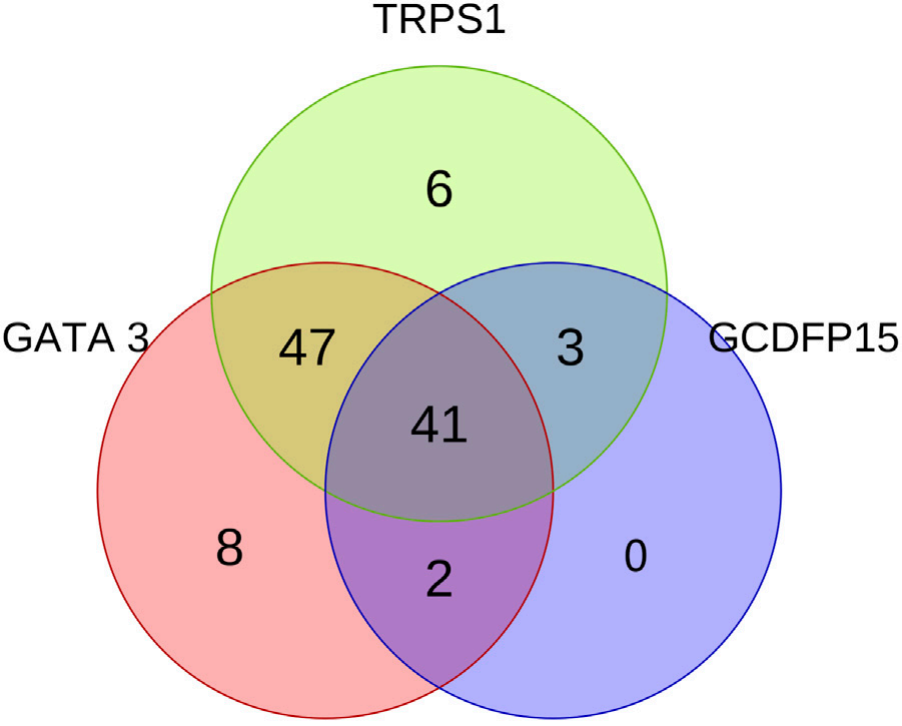
The Venn diagram illustrates the expression patterns and interrelationships of GATA3, TRPS1, and GCDFP15 in all ER-negative or low-expressing DMBC. The three circles in the diagram represent the expression of GATA3 (red), TRPS1 (green), and GCDFP15 (blue), respectively.

Among the 107 cases of DMBC, the most common metastatic sites were cervical lymph nodes (38.32%, 41/107), lung (18.69%, 20/107), and liver (11.21%, 12/107). The positive expression rates of TRPS1, GATA3, and GCDFP-15 in DMBC were 90.65% (97/107), 91.59% (98/107), and 42.99% (46/107), respectively (Table 1). There was no significant difference in the expression rate and intensity of the first two (*p* = 0.929), but both were higher than GCDFP-15 (*p* < 0.05). Among these, 6 cases showed positive expression for TRPS1 while GATA3 and GCDFP-15 were negative (Figure 1); 8 cases showed positive expression for GATA3 while TRPS1 and GCDFP-15 were negative (Figure 2). No cases were negative for both TRPS1 and GATA3 while GCDFP-15 was positive. In six cell block cases, the positive rates of TRPS1, GATA3, and GCDFP-15 were 100% (6/6), 66.67% (4/6), and 66.67% (4/6), respectively. 66.67% of the cell block samples showed moderate to strong positive expression of TRPS1 (Table 2). These three markers demonstrated differential expression patterns in special types of DMBC (Table 3). 24.73% (23/93) of cases showed a change in the ER status between the primary tumor and DMBC (from positive to negative or low expression). The HER2 expression of the primary tumor in these cases was also variable (3 cases 0, 9 cases 1+, 5 cases 2+, 7 cases 3+). HER2 expression in the primary tumor was detected in 92.52% (99/107) of the cases, of which HER2 3+ accounted for the highest proportion (36.37%, 37/99). Majority of cases with HER2 3+ expression in primary tumors also exhibited 3+ expression in the metastatic foci (83.78%, 31/37). In addition, we also summarized and analyzed the clinical data of the primary tumors in these metastatic cases. Among the 107 cases, clinical data for the primary tumor was available in 93 cases (86.92%), with 51.61% of the primary tumors located on the left side and 48.39% on the right side. The time from detection of the breast tumor to confirmation of distant metastasis ranged from 4 to 230 months, with a median of 46 months. Simultaneously, our analysis of the specificity among the three markers revealed no statistically significant difference between TRPS1 and GATA3 (*p = 1*), though both demonstrated significantly higher specificity than GCDFP-15 (*p* < 0.05) (Supplementary Tables S1–S3). Notably, no cases exhibited concurrent negativity for both GATA3 and TRPS1, thus indicating 100% specificity (107/107) when these two markers were used in combination (Figure 3).

**TABLE 1:** Expression of TRPS1, GATA3, and GCDFP-15 in all ER-negative or low-expression DMBC.

Negative (n, %)		Positive (n, %)			Total	*p-value*
		Weak positive	Moderate positive	Strong positive		
TRPS1						
	10 (9.35%)	7 (6.54%)	19 (17.75%)	71 (66.36%)	107	*p1 =* 0.929
GATA3						
	9 (8.41%)	7 (6.54%)	21 (19.63%)	70 (65.42%)	107	*p2* < 0.05
GCDFP-15						
	61 (57.01%)	13 (12.15%)	12 (11.21%)	21 (19.63%)	107	*P3* < 0.05

*p1* represents the expression difference between TRPS1 and GATA3, *p2* represents the expression difference between TRPS1 and GCDFP-15, *p3* represents the expression difference between GATA3 and GCDFP-15.

**TABLE 2:** Expression of TRPS1, GATA3, and GCDFP-15 in cell block specimens of DMBC.

Negative (n, %)		Positive (n, %)			Total
		Weak positive	Moderate positive	Strong positive	
TRPS1					
	0	2 (33.33%)	1 (16.67%)	3 (50%)	6
GATA3					
	2 (33.33%)	2 (33.33%)	2 (33.33%)	0	6
GCDFP-15					
	2 (33.33%)	1 (16.67%)	0	3 (50%)	6

**TABLE 3:** Expression of TRPS1, GATA3, and GCDFP-15 in special types of DMBC.

Type	NO.	ER	GATA3	TRPS1	GCDFP15
Invasive lobular carcinoma	1	-	1+	-	3+
invasive carcinoma with apocrine differentiation	1	-	3+	3+	2+
invasive carcinoma with neuroendocrine differentiation and focal invasive micropapillary carcinoma differentiation	1	-	3+	2+	1+
Metaplastic carcinoma (matrix-producing carcinoma)	1	-	2+	3+	-
Metaplastic carcinoma (mixed invasive ductal carcinoma- adenosquamous carcinoma)	1	-	3+	3+	-
Metaplastic carcinoma (mixed invasive ductal carcinoma-squamous carcinoma)	1	-	2+	-	1+

## Discussion

Metastasis is a late event in many malignancies and a major factor leading to patient mortality. Identifying the origin of metastatic tumors and implementing targeted therapies is crucial. Most breast cancers are ER positive, and when combined with the patient’s history or the presence of a breast nodule, a diagnosis can be made clearly. However, some breast cancers are ER-negative or present with metastatic nodules as the first symptom. In some cases, the hormone receptor status of the metastatic lesions may change, making diagnosis more difficult. The currently commonly used breast-origin markers have certain limitations. Moreover, their positivity rates are low in TNBC, and their diagnostic value is limited for ER-negative or low-expression cases. Therefore, it is necessary to explore new immunohistochemical markers as assistant. In 2004, Chang et al unexpectedly discovered high expression of TRPS1 mRNA in both breast cancer and normal breast tissue while investigating the expression of TRPS1 mRNA in prostate cancer [20]. Over the past decade, TRPS1 has been studied as a new breast cancer marker. Recent studies reveal that TRPS1 shows a positivity rate exceeding 90% in most special types of breast cancer, including 100% positive expression in adenoid cystic carcinoma and secretory carcinoma. In metastatic special types of breast cancer, the positivity rate is 92.86% (26/28). In the TNBC subtype, the positivity rate of TRPS1 is 90.4%, and its expression is negatively correlated with AR [21]. These findings support TRPS1 as a highly sensitive marker for special types of breast cancer. Moreover, in CK5-positive TNBC, the positivity rate of TRPS1 is significantly higher than that of SOX10, GATA3, mammaglobin, and GCDFP15 [22]. Ding et al summarized the application value of GATA3, GCDFP-15, mammaglobin, TRPS1, and SOX10 in differentiating breast metastases from metastases of other origins, finding that approximately half of TNBC cases were negative for all three markers (GATA3, GCDFP-15, mammaglobin). Therefore, when encountering metastatic triple-negative cancer from other organs, without thoroughly reviewing the patient’s clinical data, negative expression of these markers cannot exclude the possibility of TNBC metastasis [23]. TRPS1 is highly expressed in TNBC and can effectively compensate for the relatively low positivity rate of GATA3. Therefore, it is recommended to use both markers together for complementary diagnostic value. Previous studies on distant metastatic cancers included relatively few cases [14, 21]. Our research results showed some differences compared to previous studies.

In our study of 107 cases of ER-negative/low expression DMBC, GATA3 still showed a relatively high positivity rate, and the expression of TRPS1 was similar to that of GATA3 (90.65% vs. 91.59%). In a few cases, TRPS1 was positive while GATA3 was negative, and *vice versa*. In our study, no cases were found to be negative for both GATA3 and TRPS1, indicating that their combined use offers extremely high specificity and can better support the identification of the breast origin in such metastatic lesions.

Some studies have also compared the expression differences of TRPS1 and GATA3 in cytological samples. The study by Abdelwahed et al suggested that in 17 cases of metastatic TNBC cytological samples, the positivity rate of TRPS1 was higher than that of GATA3 (100% vs. 64.71%) (results were consistent in body fluids and fine needle aspiration specimens). The average percentage of positive cells for TRPS1 was also higher than that for GATA3 (84.4% vs. 52.6%) [16]. Two studies on TRPS1 in metastatic breast cancer cytological samples indicated that the positivity rates of TRPS1 and GATA3 were similar (7/9 vs. 6/9; 5/7 vs. 5/7) [17, 24]. Bradt et al collected six TNBC cytological specimens, and the results showed that the positive rates of TRPS1 and GATA3 were 66.7% and 100% respectively [25]. Baban et al compared breast cancer pleural effusion specimens and fine needle aspiration specimens from mesotheliomas and confirmed that TRPS1 was more specific than GATA3 in confirming breast origin, as both markers were 100% positive in pleural effusion, but there was a significant difference in positivity rates in mesotheliomas (5% vs. 84%) [26]. There are six ER-negative cell blocks in our cases, all cases showed positive TRPS1 expression, while the positivity rates for GATA3 and GCDFP-15 were same (both 66.67%). The moderate to strong positivity rate of TRPS1 was 66.67%, higher than that of GATA3 and GCDFP-15 (33.33% and 50%). These results support that TRPS1 has greater advantages over the latter two markers in metastatic TNBC cytological samples. Due to the small sample size of cytological specimens for metastatic TNBC, the expression of these markers may vary. Nevertheless, TRPS1 and GATA3 could be a reliable panel to determine the breast origin of metastatic cancer.

When immunohistochemical markers are used to suggest the origin of a tumor, special attention must be paid to the specificity of each marker. Previous studies have shown that TRPS1 exhibits limited expression in certain tumors, such as lung squamous cell carcinoma, ovarian serous carcinoma, ovarian non-serous carcinoma, and salivary duct carcinoma. TRPS1 is rarely expressed in some types of tumors, including urothelial carcinoma, lung adenocarcinoma, pancreatic cancer, colorectal and gastric adenocarcinoma, and melanoma [10, 14]. Nevertheless, some aberrant TRPS1 expression has been observed. Rammal et al. used tissue microarrays to analyze TRPS1 expression in various tumor, including breast cancer, endometrial cancer, and ovarian cancer, etc. [27]. They found that while the positive expression rate of TRPS1 in TNBC (triple-negative breast cancer) was nearly 90%, weaker expression was also observed in 71% of endometrial cancers. Furthermore, under various experimental conditions, the specificity of TRPS1 for breast cancer or TNBC specifically was consistently less than 70%. In addition, Lung adenocarcinoma and ovarian serous carcinoma can also exhibit diffuse positive expression of TRPS1 [10, 23]. The study by Bachert et al. found that TRPS1 had expression rates of 31% and 27% in prostate cancer and bladder urothelial carcinoma, respectively, which might be associated with the use of different clones of antibody [28]. In rare cases, primary or recurrent TNBC cases with diffuse TRPS1 positivity may show focal positive expression of TTF-1, PAX8, and CDX2 [23]. Additionally, most ER-/PR-/AR+ invasive carcinomas with apocrine differentiation exhibited negative expression of TRPS1 and GATA3. Therefore, the absence of TRPS1 expression does not exclude a breast origin for the tumor [29]. For mesenchymal tumors of the breast, Wang et al. found that TRPS1 is highly expressed in phyllodes tumors, chondrosarcomas, and extraskeletal osteosarcomas [15]. Recent research by Pancsa et al. showed that 60% of angiosarcomas exhibit TRPS1 positivity [30], posing a new challenge for the differential diagnosis of ER-negative breast tumors. GATA3 has been well-studied and is used to assist in diagnosing metastatic tumors of breast origin. Its high expression in salivary duct carcinoma, cutaneous adnexal tumors, and T-cell lymphoma has been widely recognized [2, 23]. Recognizing the problem of specificity above can effectively reduce the risk of misinterpreting immunohistochemical staining results and prevent misdiagnosis.

In addition, multiple studies have confirmed that the positive rate of mammaglobin and GCDFP15 in breast cancer is much lower than that of TRPS1 and GATA3, especially in TNBC and basal-like breast carcinoma (the positive rates of mammaglobin and GCDFP15 are lower <35% and 16% in TNBC; 21.4% and 11.9% in basal-like carcinoma, respectively). Consequently, their values in confirming breast origin of metastatic carcinoma are limited [31]. Our results of GCDFP15 expression in ER-negative/low expression DMBC cases further confirm previous conclusions (42.99% positivity). Moreover, GCDFP-15 often shows focal or patchy cytoplasmic staining in tumor cells, which sometimes makes it difficult to distinguish from background staining [23]. There are also other markers used to suggest the origin of breast cancer, while showing its own limitations. For example, Wnt9b, FOXC1, and SOX10, and so forth. Wnt family member 9b (Wnt9b) is one of 19 Wnt family proteins and plays an important role in kidney development and in the nasal/maxillary processes [32, 33]. Its abnormal expression can influence the stability and activity of β-catenin, promoting gene stability, proliferation, metastasis, immune responses, and other processes in cancer cells, which may be associated with the invasion and metastasis of breast cancer [34]. Some reports indicate that its positivity rates in primary and metastatic breast cancer are 98.7% and 87.3%, respectively, while it is negatively expressed in urothelial carcinoma, supporting its role as a new breast cancer marker with high sensitivity and specificity. However, in TNBC, the positivity rate of Wnt9b is slightly lower, (only 83%) [35]. Another similar study including 34 cases of non-metaplastic TNBC and 67 cases of metaplastic carcinoma showed that regardless of using whole tissue sections or tissue microarrays, the positivity rate of Wnt9b exceeded 90% in the two groups, while it was 80% in the metaplastic carcinoma group. It was significantly higher than GATA3 (56%) and SOX10 (48%) but slightly lower than TRPS1 (90%) [36]. Studies on metastatic cytology suggest that Wnt9b has slightly higher specificity compared to GATA3 (93.5% vs. 70.3%) but lower sensitivity (81.3% vs. 92.7%) [34]. Therefore, in cases with ER-negative or low expression, Wnt9b still needs to be used in combination with other breast cancer markers to demonstrate its value in differential diagnosis. Currently, the number of cases in related studies is limited, and further large-scale research is necessary.

Forkhead Box C1 (FOXC1), a member of the FOX family of transcription factors, plays a crucial role in embryonic development and the progression of various tumors [37–39]. In TNBC, FOXC1 can co-regulate with L1 cell adhesion molecule (L1CAM) to promote cancer cell invasion, motility, and lung metastasis [37]. Although FOXC1 is highly expressed in 77.8% (288/370) of TNBC cases [40], its greater significance lies in its role in TNBC molecular subtyping. Positive expression of FOXC1 supports the basal-like immune-suppressed (BLIS) subtype, which is characterized by high genomic instability and elevated expression of vascular endothelial growth factor (VEGF), providing a rationale for PARP inhibitor therapy [41, 42]. However, FOXC1 has limited utility in indicating breast origin. Studies show that its sensitivity is lower than TRPS1, but it can be used alongside SOX10 to help identify the basal-like subtype of breast cancer [43, 44].

SOX10 was initially reported as a marker for neurogenic tumors and malignant melanoma [45]. Subsequent research found that it is also highly expressed in tumors with myoepithelial differentiation, as well as in metaplastic carcinoma and basal-like breast cancer. Furthermore, it can even be expressed in clear cell sarcoma, granular cell tumor, gastrointestinal stromal tumor, and glioma [23, 44, 46]. The overall positive rate of SOX10 in breast cancer ranges from 6.5% to 40%, and is only about 60% in TNBC [46]. Some researchers have demonstrated that the expression of SOX10 is inversely correlated in TNBCs [11]. Therefore, its utility in indicating breast origin is limited. Another study showed that TNBC patients with dual-negative expression of SOX10 and AR have a worse prognosis. Therefore, SOX10 is not considered a true breast cancer marker but may more likely indicate basal/myoepithelial differentiation in breast cancer. Recent studies suggest that SOX10 can influence the epithelial-mesenchymal transition (EMT) process, and its expression is related to immune responses, indicating that it could serve as a target for immunotherapy [47]. As a result, SOX10 is generally not routinely used as a marker for breast origin in clinical practice.

It is evident that diagnosing breast cancer solely based on immunohistochemical results or a single marker staining result carries certain risks. In the cases of our study, all patients had primary breast tumors. Some developed distant metastasis after the diagnosis and treatment of breast cancer, while others had metastatic lesions detected firstly (such as axillary lymph nodes and supraclavicular lymph nodes), followed by a breast biopsy that confirmed the presence of a primary breast tumor. Moreover, 24.73% (23/93) cases showed a change of ER status between the primary tumor and distant metastatic breast cancer (from positive to negative or low expression). Additionally, 36.37% (37/99) of cases demonstrated HER2 3+ positivity in the primary tumors, indicating that ER-negative or low-expression metastatic cases do not represent all TNBC. These factors complicate the definitive diagnosis of breast cancer metastasis, especially in cases where the primary tumor is TNBC. However, for HER2-overexpressed cases, 83.78% (31/37) of the metastatic lesions retained HER2 3+ expression, which partially supports the breast origin of the metastatic carcinoma. By combining histological morphology, clinical data, and a set of immunohistochemical marker staining results, the final diagnosis of breast cancer metastasis was made.

## Conclusion

The ER status between the primary tumor and DMBC can change, so ER-negative or low expression cannot completely exclude the possibility of breast cancer metastasis. Our results show that, whether in histological or cytological specimens, TRPS1 is equally effective as GATA3 in confirming breast origin. Both markers have excellent complementary effects and are superior to GCDFP-15. The combined application of TRPS1 and GATA3 is the best method to determine breast origin of ER-negative or low-expression distant metastatic cancers.

## Data Availability

The original contributions presented in the study are included in the article/Supplementary Material, further inquiries can be directed to the corresponding author.
