# Supplementary material for: Application of TRPS1 in ER-negative or low expression distant metastatic breast carcinoma
Source: Pathol Oncol Res. 2025 Aug 1;31:1612138. doi: 10.3389/pore.2025.1612138 (PMC12355182; doi:10.3389/pore.2025.1612138)
Supplement: Supplementary file 1 [file Supplementaryfile1.docx]

**SUPPLEMENTARY TABLE S1.**

Comparison of the Specificity of GATA3 and TRPS1 in All ER-Negative or Low-Expression DMBC

| TRPS1  GATA3 | Positive | [Negative](http://www.baidu.com/link?url=JmSVv59KLNFhnzNhQvzdyDJEVPyTUfWxpfYpZttDDNDJiXJef_aDn3ErzN-ur39bHLTCbpLndGfBklTswMrbz_) | Total |
| --- | --- | --- | --- |
| Positive | 88 | 10 | 98 |
| [Negative](http://www.baidu.com/link?url=JmSVv59KLNFhnzNhQvzdyDJEVPyTUfWxpfYpZttDDNDJiXJef_aDn3ErzN-ur39bHLTCbpLndGfBklTswMrbz_) | 9 | 0 | 9 |
| Total | 97 | 10 | 107 |

*p*=1, *κ*=-0.097

**SUPPLEMENTARY TABLE S2.**

Comparison of the Specificity of GATA3 and GCDFP15 in All ER-Negative or Low-Expression DMBC

| GCDFP15  GATA3 | Positive | [Negative](http://www.baidu.com/link?url=JmSVv59KLNFhnzNhQvzdyDJEVPyTUfWxpfYpZttDDNDJiXJef_aDn3ErzN-ur39bHLTCbpLndGfBklTswMrbz_) | Total |
| --- | --- | --- | --- |
| Positive | 43 | 55 | 98 |
| [Negative](http://www.baidu.com/link?url=JmSVv59KLNFhnzNhQvzdyDJEVPyTUfWxpfYpZttDDNDJiXJef_aDn3ErzN-ur39bHLTCbpLndGfBklTswMrbz_) | 3 | 6 | 9 |
| Total | 46 | 61 | 107 |

*p*<0.001, *κ*=0.029

**SUPPLEMENTARY TABLE S3.**

Comparison of the Specificity of TRPS1 and GCDFP15 in All ER-Negative or Low-Expression DMBC

| GCDFP15  TRPS1 | Positive | [Negative](http://www.baidu.com/link?url=JmSVv59KLNFhnzNhQvzdyDJEVPyTUfWxpfYpZttDDNDJiXJef_aDn3ErzN-ur39bHLTCbpLndGfBklTswMrbz_) | Total |
| --- | --- | --- | --- |
| Positive | 44 | 53 | 97 |
| [Negative](http://www.baidu.com/link?url=JmSVv59KLNFhnzNhQvzdyDJEVPyTUfWxpfYpZttDDNDJiXJef_aDn3ErzN-ur39bHLTCbpLndGfBklTswMrbz_) | 2 | 8 | 10 |
| Total | 46 | 61 | 107 |

*p*<0.001, *κ*=0.077
